# Identification of a novel IL-5 signaling pathway in chronic pancreatitis and crosstalk with pancreatic tumor cells

**DOI:** 10.1186/s12964-020-00594-x

**Published:** 2020-06-17

**Authors:** Sarah B. Gitto, Jordan M. Beardsley, Sai Preethi Nakkina, Jeremiah L. Oyer, Kathryn A. Cline, Sally A. Litherland, Alicja J. Copik, Amr S. Khaled, Na’im Fanaian, J. Pablo Arnoletti, Deborah A. Altomare

**Affiliations:** 1 Burnett School of Biomedical Sciences, College of Medicine, University of Central Florida, 6900 Lake Nona Blvd., Orlando, FL 32827 USA; 2 Present Address: Department of Pathology and Laboratory Medicine, Abramson Cancer Center, Perelman School of Medicine, University of Pennsylvania, Philadelphia, PA 19104 USA; 3 AdventHealth Cancer Institute, Orlando, FL 32804 USA; 4 Orlando Veteran’s Affairs Medical Center, Orlando, FL 32827 USA; 5 AdventHealth Lab, Orlando, FL 32804 USA; 6 AdventHealth Cancer Institute and Institute for Surgical Advancement, Orlando, FL 32804 USA

**Keywords:** Pancreatic ductal adenocarcinoma, Pancreatitis, IL-5, IL-5R, Eosinophils

## Abstract

**Background:**

While inflammation is associated with pancreatic cancer, the underlying mechanisms leading to cancer initiation are still being delineated. Eosinophils may promote or inhibit tumor growth, although the specific role in pancreatic cancer has yet to be determined. Eosinophil-supporting cytokine interleukin-5 and receptor are likely to have a role, but the significance in the pancreatic cancer microenvironment is unknown.

**Methods:**

Genetically engineered Akt1^Myr^/KRas^G12D^ and KRas^G12D^ mice were used to model changes induced by chronic inflammation. Tissue samples were collected to analyze the tumor microenvironment and infiltration of immune cells, whereas serum was collected to analyze cytokine and amylase activity in the inflammatory model. The expression of IL-5R and the effects of IL-5 were analyzed in human and murine tumor cells.

**Results:**

Compound Akt1^Myr^/KRas^G12D^ mice, compared to single KRas^G12D^ or Akt1^Myr^ mice, exhibited increased tissue damage after repeat inductions of inflammation, and had accelerated tumor development and metastasis. M2 macrophages and newly identified eosinophils co-localized with fibrotic regions rather than infiltrating into tumors, consistent with immune cell privilege. The majority of eosinophils found in the pancreas of Akt1^Myr^/KRas^G12D^ mice with chronic inflammation lacked the cytotoxic NKG2D marker. IL-5 expression was upregulated in pancreatic cells in response to inflammation, and then diminished in advanced lesions. Although not previously described in pancreatic tumors, IL-5Rα was increased during mouse pancreatic tumor progression and expressed in human pancreatic ductal adenocarcinomas (7 of 7 by immunohistochemistry). IL-5 stimulated tumor cell migration and activation through STAT5 signaling, thereby suggesting an unreported tumor-promoting role for IL-5Rα in pancreatic cancer.

**Conclusions:**

Chronic inflammation induces increased pancreatic cancer progression and immune cells such as eosinophils are attracted to areas of fibrosis. Results suggest that IL-5 in the pancreatic compartment stimulates increased IL-5Rα on ductal tumor cells to increase pancreatic tumor motility. Collectively, IL-5/IL-5Rα signaling in the mouse and human pancreatic tumors microenvironment is a novel mechanism to facilitate tumor progression.

Additional file 1:**Video Abstract**

## Background

With a five-year survival rate of only 9 %, pancreatic ductal adenocarcinoma (PDAC) is one of the most lethal of all major tumor types. Here we will focus on the desmoplastic microenvironment and pro-tumor characteristics, which are postulated to make current chemotherapeutic or immunotherapy treatment challenging [1].

Acute insult to the pancreas results in an inflammatory environment, which promotes anaplasia of the pancreatic cells to a progenitor-like state, allowing proliferation of cells and tissue remodeling to repair the damage. Acinar cells undergo a transition to become duct like, and when the acute inflammation is resolved, they are able to transition back to their acinar phenotype [2]. Failure to resolve acute inflammation leads to chronic inflammation, which will stabilize acinar-to-ductal metaplasia (ADM) [2]. Thus, chronic pancreatitis is an established factor for pancreatic cancer [3]. Delineating the role of this risk factor and how it alters the tissue microenvironment to promote tumor onset may lead to new treatment options.

Pancreatic fibrosis and a desmoplastic phenotype are common characteristics of chronic pancreatitis and pancreatic cancer. Inflammation causes quiescent pancreatic stellate cells (PSCs) to become activated and transform into fibroblast-like cells [4]. PSCs potentiate changes to the extracellular matrix, such as the secretion of collagen and periostin (osteoblast-specific factor-2), while an increasingly fibrotic stroma reduces the ability of chemotherapeutics to penetrate the tumor [5]. Activated PSCs also produce cytokines that recruit immune cells that further perpetuate the production of fibrosis and promote tumor formation. During chronic inflammation, macrophages are recruited to the activated stroma and become polarized by local interleukins towards an alternatively activated tumor associated macrophage (M2) phenotype. M2 macrophages stimulate the production of collagen through the expression of arginase-1 and L-proline [6].

Eosinophils also increase collagen production by fibroblast cells via release of eosinophil peroxidase (EPO) [7]. In cancer, the presence of tumor-associated tissue eosinophilia (TATE) or eosinophil degranulation has been correlated with improved prognosis [8]. In some tumors, eosinophils adopt cytotoxic properties against tumor cells via expression of cytotoxic markers including NKG2D. NKG2D neutralizing antibody was previously shown to reduce activated eosinophil toxicity against hepatocellular carcinoma cells [9]. In contrast, eosinophil infiltration has been linked to poorer prognosis in other tumor types including, Hodgkin lymphoma, oral squamous cell carcinoma, and cervical carcinoma [10, 11]. Eosinophils stimulate T-cell proliferation and T helper (T_h_) 2 cells polarization via T_h_1 apoptosis, as well as shift the microenvironment towards T-regulatory cell accumulation [12, 13]. Although eosinophils have been previously reported in pancreatic cancer [14, 15], their role has yet to be defined.

IL-5 is a cytokine that is responsible for the recruitment, proliferation, maturation and activation of eosinophils [16–20]. IL-5 enhances eosinophil adhesion and migration on periostin [21]. Recent reports indicate that IL-5 has a role in accumulation of pancreatic fibrosis. Mice lacking IL-5 have reduced eosinophil infiltration and reduced collagen formation after cerulein injections compared to mice with an intact IL-5 gene [22]. IL-5 also stimulates growth and differentiation of B-cells, and can prime mast cells by increasing their production of pro-tumor and pro-fibrotic cytokines including TNF-α, IL-5, IL-13, MIP-1α, and granulocyte- macrophage colony-stimulating factor (GM-CSF) [23]. In addition, IL-5 promotes collagen synthesis and in the presence of pancreatic stroma may induce differentiation of M2 macrophages [24]. The receptor IL-5Rα has been characterized in the context of immune cell signaling, but its expression on other tissue types has not been well characterized.

A recent study reports that cerulein induced murine models of pancreatitis increases eosinophil accumulation. Mice lacking GATA, a transcription factor required for eosinophil development, has reduced acinar remodeling, and reduced mast cell and collagen accumulation, suggesting a role for eosinophils in chronic pancreatitis [22]. This report aims to understand the complex interplay of a fibrotic microenvironment and the role that pro-tumor immune cell infiltration has on the progression of pancreatic cancer in the context of chronic pancreatitis. We previously reported accelerated PDAC formation in genetically engineered mice with constitutively active myristoylated Akt1 (Akt1^myr^) and active mutant KRas^G12D^ (Akt1^Myr^/KRas^G12D^) [25]. The objective for this study was to determine if repeated injections of the cholecystokinin analog cerulein (CER), which can experimentally induce chronic pancreatitis, could accentuate tumor development in Akt1^Myr^/KRas^G12D^ mice over that of untreated controls or cerulein-treated KRas^G12D^ mice. At comparable times across the different treatment groups, Akt1^Myr^/KRas^G12D^ mice exhibited the most severe pancreatic tissue remodeling and damage response. Here, a dominant type II immune response and extensive fibrosis likely facilitated infiltration of B-cells and granulocytes, such as mast cells and eosinophils, in the response to chronic inflammation. Findings led to the discovery of a new signaling mechanism of crosstalk between IL-5 expression in the pancreatic microenvironment and IL-5Rα on pancreatic tumor cells, which may be a novel targetable pathway in pancreatic tumor progression.

## Methods

**Fig. 1:**
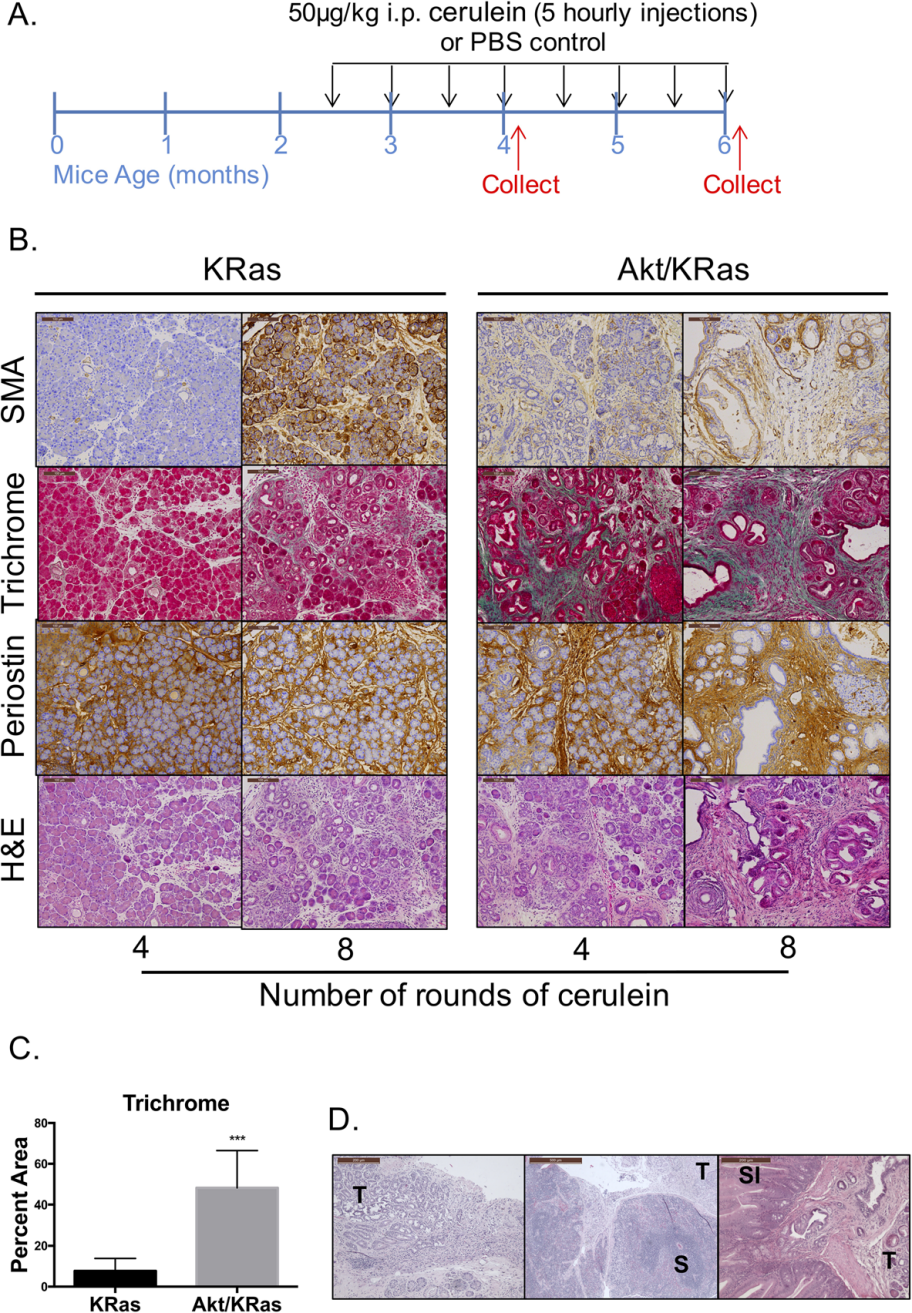
Akt1^Myr^/KRas^G12D^ mice show profound stromal activation and tissue remodeling with chronic inflammation. **a** Schematic of cerulein (CER) injection strategy shows that all genotypes were injected i.p. hourly for 5 h with PBS or 50 μg/kg CER bi-weekly for 3 months. Mice were euthanized 48 h (for histology) and 16 h (for flow cytometry) after the 4th and 8th injections (indicated by the red arrows). **b** Histology of pancreatic tissue for KRas^G12D^ and Akt1^Myr^/KRas^G12D^ mice injected with 4 rounds of CER injections (4-month-old) or 8 rounds of CER injections (6-month-old). Smooth muscle actin (α-SMA; brown) for activated PSCs near fibrotic regions. Trichrome staining for acinar tissue (red) & collagen-rich matrix (green-blue) in fibrotic areas. Periostin (brown) expression correlating to regions of activated PSCs and collagen. Images were acquired at 10x magnification and the scale bar denotes 100 μm. **c** Quantification of percent area of trichrome staining in Akt1^Myr^/KRas^G12D^ and KRas^G12D^ after 8 rounds of CER (*n* = 7 mice, 3 to 8 random fields were analyzed per mouse). **d** Akt1^Myr^/KRas^G12D^ mice with chronic inflammation are prone to developing tumors that metastasize. H&E of Akt1^Myr^/KRas^G12D^ pancreas tumors (T) and/or spleen (S) or small intestine (SI) metastasis after cerulein from PDAC diagnosed mice in Table S1

Tissues and serum were collected from mice after four or eight rounds of cerulein injections as outlined in Fig. 1a and Methods. Tissues were formalin fixed and processed for histological analysis by a certified pathologist (A.S.K.). In subsequent studies, pancreas was collected after four rounds of cerulein injections and processed for immune cell analysis by flow cytometry. Serum collected from mice was used for analysis of cytokines and amylase activity. For human tissue analysis, tissues were collected at the time of surgery from pancreatic cancer patients. Tumor tissue and nonadjacent normal tissues were collected and prepared for analysis on the day of resection. Patients were diagnosed (Table S2) and IL-5Rα staining was confirmed by a certified pathologist (N.F.).

### Clinical samples

Tissues were collected at the time of surgery from consenting patients at AdventHealth, previously Florida Hospital, under IRB approved protocol 507,397 and used in experiments only as HIPAA de-identified tissues.

### Breeding and genotyping

All mice were housed and handled in accordance with protocols approved by the UCF Institutional Animal Care and Use Committee, an Association for Assessment and Accreditation of Laboratory Animal Care International accredited facility. Transgenic mice with activation of KRas^G12D^ (Pdx-Cre;LSL-KRas^G12D^) were mated with transgenic mice with activation of Akt1^Myr^ (Pdx-tTA;TetO-MyrAkt1). Mice were genotyped as previously described [25].

### Experimental pancreatitis

Wild type (Wt), Akt1^Myr^, KRas^G12D^, and Akt1^Myr^/KRas^G12D^ mice at 10–12 weeks of age were divided into PBS treated and cerulein treated groups. Mice were dosed intraperitoneally with 50 μg/kg cerulein (American Peptide Company, Sunnyvale, CA) once each hour for 5 hours (one series), every other week, for up to eight total injection series. Control mice from all genotypes received comparable injections of 0.9% sodium chloride (saline). Serial blood collections from the submandibular vein, was collected ~ 16 h post injections, and serum was used for cytokine and amylase activity analysis. Mice were monitored daily for diarrhea, signs of distress, and periorbital exudates. Mice exhibiting signs of distress were euthanized according to American Veterinary Medical Association guidelines.

At the termination of the study, mice were sacrificed, and tissues were collected at approximately 16 or 48 h post the last injection following the fourth or eighth rounds of injections. Mice were euthanized by cardiac puncture under isoflurane and secondarily by cervical dislocation. Pancreata, liver, small intestine, and spleen were collected and fixed in 10% buffered formalin for histological analysis or in RPMI to be further processed for flow cytometry analysis.

### Immunohistochemistry

Specimens were fixed in 10% neutral buffered formalin (Surgipath Leica, Buffalo Grove, IL) and paraffin embedded prior to sectioning with a rotary microtome (5um; Leica). Sections attached to charged microscope slides were dried at 65 °C for 30 min in a hybridization oven. Slides were stained for hematoxylin and eosin (H&E) reagents (Surgipath). Staining with Trichrome or Combined Eosinophil-Mast Cell (C.E.M.; American MasterTech, Lodi, CA) was performed as per manufacturer’s instructions.

Immunohistochemistry using Polymer Refine Detection reagents was performed using the Bond-Max Immunostainer (Leica). The tissue was dehydrated, and cover slipped. Antigen retrieval was optimized using sodium citrate, pH 6.0 or EDTA, pH 9.0 (Leica), and all antibody staining was optimized in relationship to no primary control staining. Primary antibodies used for this study include smooth muscle actin (α-SMA; Abcam), Periostin (AdipoGen, San Diego, CA), macrophage marker F4/80 (Thermo Fisher Scientific, Waltham, MA), M2 macrophage marker CD206 (mannose receptor; Bioss, Woburn, MA), eosinophil marker PRG2 (Bioss), B-cell marker CD19 (Bioss), IL-5 (Santa Cruz, Dallas, TX), and IL-5Rα (Bioss). Images were taken using a Leica DM 2000 microscope with 5X, 10X, or 40X objectives. For immune cells quantification, three to five images per slide were imaged at 40x using BZ-X810 All in One fluorescence microscope (Keyence Corporation of America, Itasca, IL). Images were analyzed with QuPath software to determine the average number of cells per image [26].

### Histological analysis of chronic pancreatitis and PanIN lesions

One hundred twenty-four mice after eight rounds of cerulein or PBS injections were histologically analyzed for pancreatitis and staged based on their tumor progression. Tissues were diagnosed with no pancreatitis, chronic severe pancreatitis (samples with > 20% of the acinar area infiltrated with mononuclear cells and fibrosis) or acute pancreatitis (infiltration of mononuclear cells without fibrosis). Upon histological assessment, mice were deemed to have incidental acute focal, mild pancreatitis as defined by samples with < 5% of acinar tissue infiltrated with mononuclear cells and replaced by fibrosis.

### Collagen staining and quantification

Samples were stained using One-Step green and red Trichrome Stain Kit. Three to eight random fields were analyzed per mouse depending on the size of the cross section of pancreas obtained. Area of collagen was determined per field using ImageJ, and the average area per mouse was calculated (*n* = 7 representative mice).

### Flow cytometry

After the fourth series of cerulein injections, mice were euthanized, and the pancreas was collected in cold PBS. For Akt1^Myr^ and Wt pancreas where there was minimal fibrosis, the pancreas was perfused with RPMI to dissociate immune cells from inside the tissue. For KRas^G12D^ and Akt1^Myr^/KRas^G12D^ studies with a higher incidence of fibrosis, the GentleMACs Dissociator with the Tumor Dissociation Kit (Miltenyi Biotec, Auburn, CA) was used to dissociate tissue. Cells were then centrifuged at 300 x g, washed with cold PBS and resuspended with antibodies diluted in staining buffer. For macrophage and T cell phenotype analysis, after the cells were incubated with extracellular marker antibodies, the cells were fixed and permeabilized using the Fixation/Permeabilization Solution Kit (BD Biosciences, San Jose, CA), per manufacturer’s instructions. Cells were then incubated with antibodies against intracellular markers, washed and resuspended in staining buffer. A total lymphocyte analysis in Akt1^Myr^ and Wt was performed by analysis of various immune cell surface markers including CD45 (total lymphocytes), CD3 (T-cells), CD11b (monocytes), Ly6G (myeloid cells). The macrophage phenotype panel included CD45, F4/80, MHCII, CD206, and iNOS. The eosinophil panel included CD45, F4/80, CD11c, CD193, SiglecF, and NKG2D. The T cells phenotype panel included CD45, CD3, CD4, and IL-4, IFNγ. Antibodies were purchased form eBiosciences (San Diego, CA). Samples were analyzed using a BD FACSCanto flow cytometer (BD Biosciences) or CytoFLEX flow cytometer (Beckman Coulter, Pasadena, CA), and data was processed with Flowlogic (Inivai Technologies, Mentone, Australia), or FlowJo (BD Biosciences) software.

### In situ hybridization

In situ hybridization to visualize single RNA molecules per cell was performed using RNAscope Assay technology (Advanced Cell Diagnostics, Hayward, CA). The assay was performed per manufacturer’s instructions and published methods [27]. Tissue slides from mouse models with cerulein induced tissue damage and PBS controls were tested.

### Cytokine bead array

Exsanguination by cardiac puncture was performed on mice ~ 48 h after the eighth dose of cerulein. Serum was separated from blood cells by centrifugation at 2000 x g for 15 min in serum separating tubes (BD Biosciences). Quantitative analysis for IL-5 was performed using the BD Biosciences Mouse IL-5 Flex Set, Cytometric Bead Array, per the manufacturer’s instructions. Samples were analyzed using a CytoFLEX flow cytometer. Raw values mean intensity fluorescent values (MFI) were acquired using Flowlogic software, and data was processed using Microsoft Excel and GraphPad Prism.

### Amylase activity assay

Blood serum was collected from Akt1^Myr^/KRas^G12D^, KRas^G12D^, and Wt mice at 12 weeks of age, before the study began as a baseline, and ~ 16 h after the sixth round of cerulein or PBS as a control. Amylase activity assay and analysis (Abcam, Cambridge, UK) was performed per manufacturer’s instructions. Sample absorbance was read at 405 nm in a colorimetric plate reader every 5 min for 1 h. Amylase activity was calculated per milliliter of serum from a standard curve.

### Transwell assay

Transwell pore size and cell number were optimized for both cell lines. 8 × 10^5^ murine pancreatic 533 cells and 1 × 10^6^ human pancreatic L3.6pl cells were placed in the top chamber of a 24-well transwell plate (Corning, Tewksbury, MA). Cells were incubated at 37 °C for 4 h (533 cells) or 6 h (L3.6pl cells) with media in the bottom chamber containing 0, 100 or 200 ng/mL of IL-5 cytokine (BioLegend, San Diego, CA). The insert was rinsed with PBS and a Q-tip was used to remove cells from the top chamber that did not migrate. The insert was fixed for 10 min with 4% paraformaldehyde, then washed with PBS. Inserts were stained with 1% crystal violet in 2% ethanol for 20 min. Inserts were washed thoroughly with diH_2_O and air-dried. Using a scalpel, the membranes were cut from the holder, mounted and a coverslip was applied using Permount (Thermo Fisher). The total number of cells that migrated through the transwell were counted from five random fields and averaged. After taking into account the viewing area and the total transwell area, the percent migration was calculated.

### Immunofluorescence

Tumor cells were grown overnight on glass chamber slides coated with poly-L-lysine (Thermo Fisher). Cells were washed and fixed with 4% paraformaldehyde for 20 min on ice. The cells were incubated overnight at 4 °C with a polyclonal IL-5Rα antibody (Bioss). Cells were washed and then incubated for 1 h at room temperature with an Alexa Fluor secondary antibody, Alexa Fluor 488 conjugated phalloidin and DAPI (Thermo Fisher). Cells were images using a Zeiss LSM 710 confocal microscope. For Stat5 activation studies, cells were treated with IL-5 (BioLegend) for 5 or 15 min prior to fixation and then incubated with phosphorylated Stat5 primary antibody (Cell Signaling Technology, Danvers, MA). Mean fluorescent intensity for phosphorylated Stat5 was calculated by Volocity imaging software (PerkinElmer, Waltham, MA) for nuclear phospho-Stat5 for each cell in the field (~ 7–15 cells for 5–10 fields). Data represents three individual experiments.

### Statistical analysis

When appropriate, results were reported as mean ± SD. Data were analyzed using one-way ANOVA with Dunnett’s multiple comparisons post-hoc test, or two-way ANOVA with Tukey’s multiple comparisons post-hoc test, or unpaired Students T-test, when appropriate. Statistical significance was set at * < 0.05, ** < 0.01, *** < 0.001, **** < 0.0001 (GraphPad Prism, La Jolla, CA).

## Results

### Chronic inflammation in Akt1^Myr^/KRas^G12D^ induces severe stromal damage and increases pancreatic tumor development

Pdx-Cre;LSL-KRas^G12D^ (herein referred to as KRas^G12D^ mice) and Pdx-tTA;TetO-MyrAkt1 mice (herein referred to as Akt1^Myr^ mice) were mated for production of double mutant and single mutant offspring [25]. Mice were intraperitoneally injected every other week with 50 μg/kg cerulein hourly for 5 hours, for up to eight rounds of injections (refer to methods and Fig. 1a). Control mice included wild-type and single mutant Akt1^Myr^ littermates injected with cerulein, which resulted in minimal pancreatic tissue fibrosis and low numbers of infiltrating immune cells (Figure S1). Additional controls included mice injected with PBS as a vehicle control. Wild type and Akt1^Myr^ PBS-injected mice showed no significant pathological lesions or inflammation (Figure S1). While KRas^G12D^ and Akt1^Myr^/KRas^G12D^ exhibited focal pancreatic intraepithelial neoplasms (PanINs, Figure S2A).

Histological assessment after eight rounds of cerulein revealed that 80% of the analyzed Akt1^Myr^/KRas^G12D^ mice display chronic pancreatitis with widespread fibrosis in place of acinar cells and infiltration of immune cells (Table S1, Fig. 1b), which was subsequently defined with additional histochemical assessment. Progression to PDAC was observed in the Akt1^Myr^/KRas^G12D^ mice following cerulein injections, and after 24 weeks of age. Forty percent of Akt1^Myr^/KRas^G12D^ mice were diagnosed with PDAC, two of which had terminal disease and were found dead at 23 and 24 weeks of age (Table S1). In advanced cases, the metastatic sites include the spleen, liver, and small intestine (Fig. 1d). The previously reported mean tumor latency of Akt1^Myr^/KRas^G12D^ mice was 54 weeks [25], thus induction of chronic inflammation resulted in a shift in early tumor latency in the Akt1^Myr^/KRas^G12D^ mice.

To quantitate the amount of stromal remodeling in Akt1^Myr^/Kras^G12D^ mice, we assessed total area of collagen by trichrome staining. Extensive stromal remodeling in Akt1^Myr^/KRas^G12D^ mice resulted in a 40.5% increase in total area of collagen formation compared to KRas^G12D^ mice (Fig. 1c). Moreover, immunohistochemistry detected increased expression of smooth muscle actin (SMA), a marker of activated stellate cells, and periostin deposition (Fig. 1b). After four rounds of cerulein injections, Akt1^Myr^/KRas^G12D^ mice had early stromal activation detected by SMA expression and precancerous lesions (Fig. 1b). In contrast, KRas^G12D^ mice presented with mainly localized ADM, PanIN precursor lesions, and acute inflammation as determined by focal neutrophil infiltration (Table S1). By using both mouse models with this modified cerulein injection strategy, we can analyze a fibrotic tumor microenvironment in parallel with an acute inflammatory precancerous model at various stages in order to better understand the roles of the pancreas microenvironment on tumor development.

### Tumor promoting leukocytes precede PDAC development during chronic inflammation

**Fig. 2:**
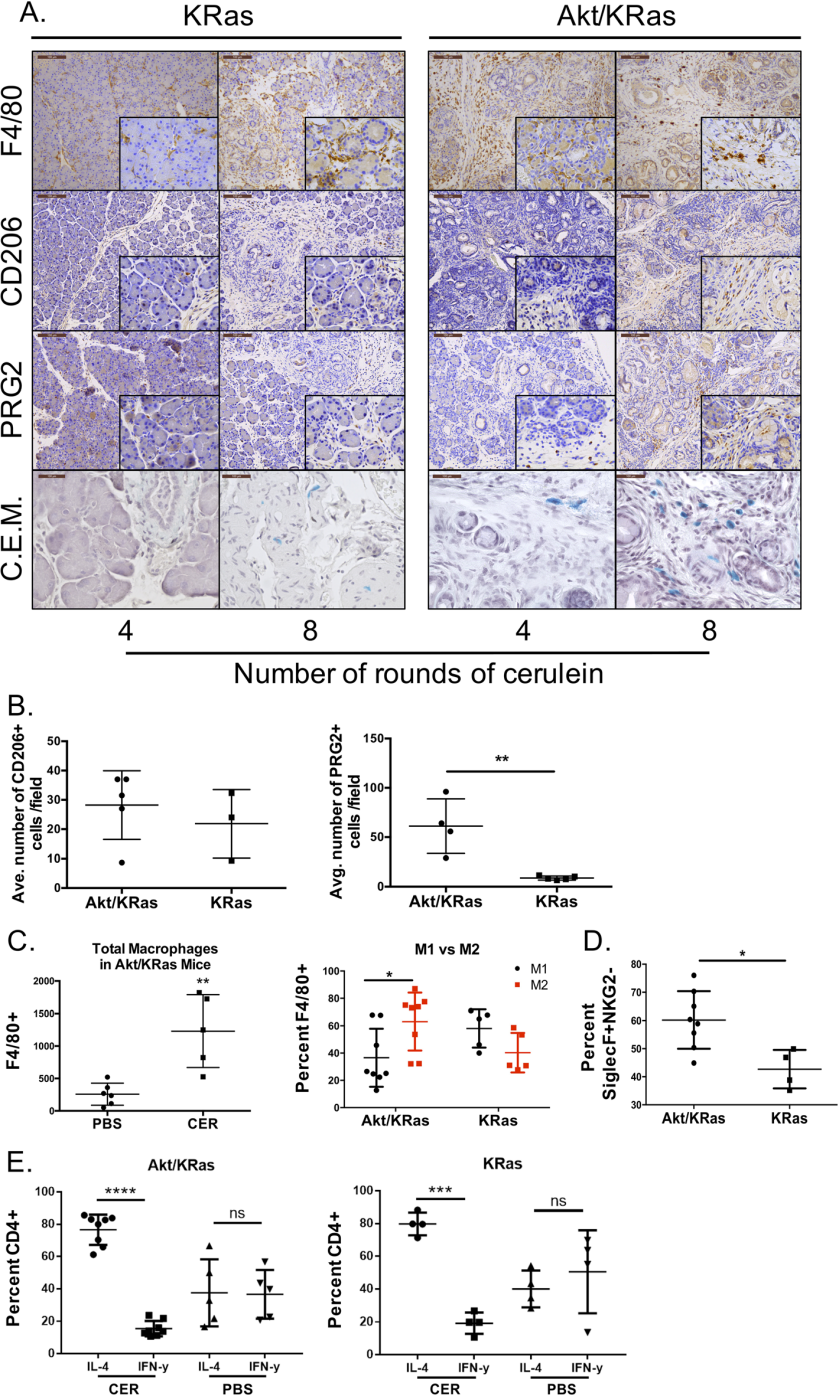
Akt1^Myr^/KRas^G12D^ mice with cerulein-induced inflammation have increased innate immune cell infiltration. **a** Immunohistochemistry for Akt1^Myr^/KRas^G12D^ and KRas^G12D^ mice injected with 4 rounds of CER (4-month-old) or 8 rounds of CER (6-month-old). Tissues with infiltrating immune cells correlate to prolonged exposure to CER and are mainly associated to collagen-rich matrix in fibrotic areas. Immunohistochemistry markers include, total macrophages (F4/80, brown), M2 macrophages (CD206, MRC1 or C-type mannose receptor 1, brown), eosinophils (PRG2, brown), and mast cells (C.E.M. staining, blue). Images were acquired at 10x magnification and the scale bar denotes 100 μm, with 40x magnification inlays. C.E.M. images were acquired at 40x. **b** Images were digitally acquired and analyzed using QuPath software for number of CD206+ (left) and PRG2+ cells (right). 3–5 slides per mouse were analyzed and the average number of cells per mouse was calculated (8 injections of CER, *n* = 3–5). **c** Flow cytometry analysis of CD45 + F4/80+ macrophages and their polarized M1 (CD45 + F4/80 + iNOS+) or M2 (CD45 + F4/80 + CD206 + MHCII) phenotype from dissociated Akt1^Myr^/KRas^G12D^ and KRas^G12D^ mouse pancreas after 4 rounds of CER or PBS. **d** Flow cytometry analysis of percent CD45 + F4/80 + CD11c-CD192 + SiglecF+ eosinophils without cytotoxic receptor NKG2D, and (E) CD45 + CD3 + CD4+ T cells with either intracellular staining for IL-4 or IFNγ from dissociated Akt1^Myr^/KRas^G12D^ and KRas^G12D^ pancreas after 4 rounds of CER injections. Flow cytometry data shown is representative data of two individual experiments (*n* = 4–8 mice per group)

To analyze the infiltration of innate immune cells during chronic inflammation, we utilized immunohistochemistry to delineate the stromal localization and also flow cytometry. Macrophages, one of the most abundant leukocytes and a well-established regulator of pancreatic cancer, are significantly increased upon induction of inflammation. Akt1^Myr^/KRas^G12D^ mice have an increased detection of general macrophage marker F4/80 following cerulein injections (Fig. 2a). After only four cerulein injections, Akt1^Myr^/KRas^G12D^ mice have significantly increased F4/80+ macrophage infiltration compared with PBS control mice (*n* = 5 and *n* = 6, respectively, Fig. 2c). Upon analysis of the total macrophage population in cerulein treated mice, Akt1^Myr^/KRas^G12D^ mice had a significant shift to M2 polarized macrophages (MHC class II^+^CD206^+^iNOS^−^). Akt1^Myr^/KRas^G12D^ mice exhibited a 33.3% increase in the ratio of MHC class II^+^CD206^+^iNOS^−^ macrophages compared to KRas^G12D^ mice (Fig. 2c). Immunohistological staining for CD206 showed that there is increased M2 macrophage infiltration in Akt1^Myr^/KRas^G12D^ compared to KRas^G12D^ and M2 macrophages primarily localized within fibrotic regions (Fig. 2a). However, after 8 cerulein injections there was no significant difference of the average number of CD206^+^ cells in pancreatic tissues of Akt1^Myr^/KRas^G12D^ and KRas^G12D^ mice (Fig. 2b, left).

Akt1^Myr^/KRas^G12D^ mice with chronic inflammation also had increased infiltration of eosinophils as determined by immunohistochemistry for PRG2 (major basic protein, MBP, Fig. 2a) and combined eosinophil and mast (C.E.M.) staining (Figure S2C). Eosinophils were primarily localized in regions of collagen-rich fibrosis of Akt1^Myr^/KRas^G12D^ mice compared to KRas^G12D^ mice presenting with acute inflammation. When comparing the staining for the eosinophil cytotoxic marker NKG2D after four rounds of cerulein injections, the majority of the eosinophils in Akt1^Myr^/KRas^G12D^ lack NKG2D expression (60.2%), where only 42.7% of eosinophils in KRas^G12D^ mice lack expression (Fig. 2d). Immunohistochemical staining showed similar results after 4 injections (Fig. 2a). After 8 injections, tissues staining showed a significant increase in PRG2^+^ cells in Akt1^Myr^/KRas^G12D^ mice compared to KRas^G12D^ mice (Fig. 2b, right; *p* = 0.0035). As results were analyzed from mice that have increased M2 polarization, it can be speculated that M2 polarization and NKG2D- eosinophils are activated by congruently expressed environmental stimulus.

Activated T helper 2 lymphocyte (T_h_2) and cytokines produced by these cells, particularly IL-5, are critical for the location and activation of eosinophils [28]. To determine the ratio of T_h_2 to T_h_1 cells, flow cytometry analysis of the pancreas after 4 injections of cerulein and PBS was performed. T_h_2 was defined as CD45^+^CD4^+^IL-4^+^ and T_h_1 was defined as CD45^+^CD4^+^INF-γ^+^ cells. Akt1^Myr^/KRas^G12D^ and KRas^G12D^ mice have significantly increased ratio of T_h_2 to T_h_1 lymphocytes, signifying a predominant T_h_2 response in both genotypes (Fig. 2e). Some Akt1^Myr^/KRas^G12D^ mice had a noticeable increase in the number of T_h_2 T-cells, however this did not reach significance across the entire cohort (data not shown).

In addition to eosinophils, there were similar trends for other cell types that are known to crosstalk with eosinophils. Eosinophils have been reported to promote B cells proliferation and support survival in vitro, and peripheral blood eosinophils correlated to the number of circulating B-cells [29]. Eosinophils also have numerous known soluble mediators and ligand/receptor interactions with mast cells, a known producer of IL-5 [30]. Immunohistochemistry for the CD19+ marker of B-cells and C.E.M. staining for mast cells showed increased cell numbers in fibrotic regions of the pancreas from Akt1^Myr^/KRas^G12D^ mice compared to KRas^G12D^ mice (Fig. 2a and S2B).

### IL-5 expression in pancreatic acinar tissue and acinar-ductal metaplastic lesions

**Fig. 3:**
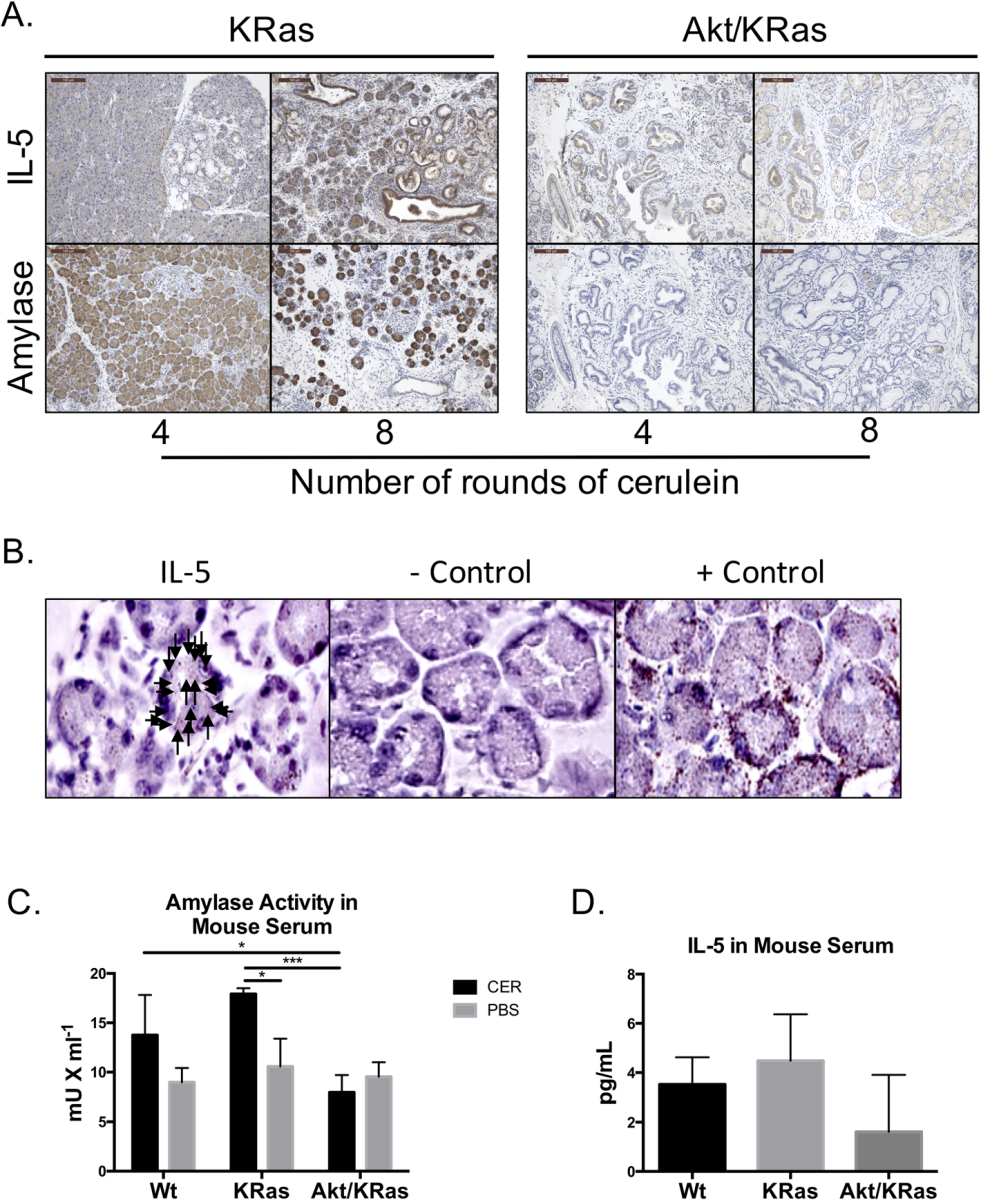
IL-5 expression in pancreas exocrine cells undergoing acinar-to-ductal metaplasia. **a** Immunohistochemistry for IL-5 and amylase in Akt1^Myr^/KRas^G12D^ and KRas^G12D^ mice injected with 4 or 8 rounds of CER. Images were acquired at 10x magnification and the scale bar denotes 100 μm. **b** Single cell in situ hybridization for IL-5 transcripts was performed using RNAscope technology. KRas^G12D^ mice with 8 rounds of CER was used for confirmation of IL-5 expression in acinar cells. DabB negative control probes and PPIB positive control probes were used for comparison. Arrows indicate positive staining for one acinar island. **c** Amylase activity measured in quantity (mU) per milliliter, from blood serum collected from Akt1^Myr^/KRas^G12D^, KRas^G12D^, and Wt mice 16 h after 6 rounds of CER and vehicle control. **d** Concentration of IL-5 in blood serum 48 h after the 8th round of CER injections

Amylase is a marker of acinar cells, and upon inflammation or damage to the acinar tissue, amylase is released into the blood stream [31]. To further characterize the damage to the tissue, amylase protein staining and serum levels were analyzed. As expected, amylase staining showed an increase in expression located in acinar and ADM cells, then a loss in pancreatic tissues with chronic pancreatitis and without remaining healthy acinar tissues (Fig. 3a). Release of circulating amylase was detectable after six rounds of cerulein injections in KRas^G12D^ mice; whereas tissue damage was well established in Akt1^Myr^/KRas^G12D^ mice with decreased detection of circulating amylase in the blood (Fig. 3c).

IL-5 is secreted by immune cells including mast cells and is an important activating cytokine for both eosinophils and B-cells. Thus, IL-5 expression in pancreatic tissues undergoing chronic inflammation was expected to be found at increasing levels associated with immune cell infiltration. In the pancreas, IL-5 is found at low mRNA expression levels (0.2 transcripts per kilobase million or TPM) in the human PDAC cell line Capan-2 and low antibody staining has been shown for IL-5 protein expression in the pancreas exocrine glandular tissue [32, 33]. Besides expression correlating to immune cells, our results showed increased expression of IL-5 in ADM cells and early PanIN lesions in KRas^G12D^ mice (Fig. 3a). However, IL5 expression was low at the same time point in Akt1^Myr^/KRas^G12D^ mice that had loss of acinar cells and advanced PanIN lesions, following a similar staining pattern to amylase. Protein analysis results were confirmed using in situ hybridization (Fig. 3b). Results indicated that IL-5 transcripts are present in cells undergoing ADM after cerulein injections. These results were surprising since significant expression levels of IL-5 are mainly reported on immune cells. There was also increased circulating IL-5 in the blood of KRas^G12D^ mice, but decreased levels in the blood of Akt1^Myr^/KRas^G12D^ mice with severely damaged pancreatic tissue (Fig. 3d). However, results did not reach statistical significance, possibly in part due to the limit of detection for pg/mL IL-5, or transient nature of IL-5 levels in peripheral blood.

### IL-5Rα activation of STAT-5 increases tumor cell migration

**Fig. 4:**
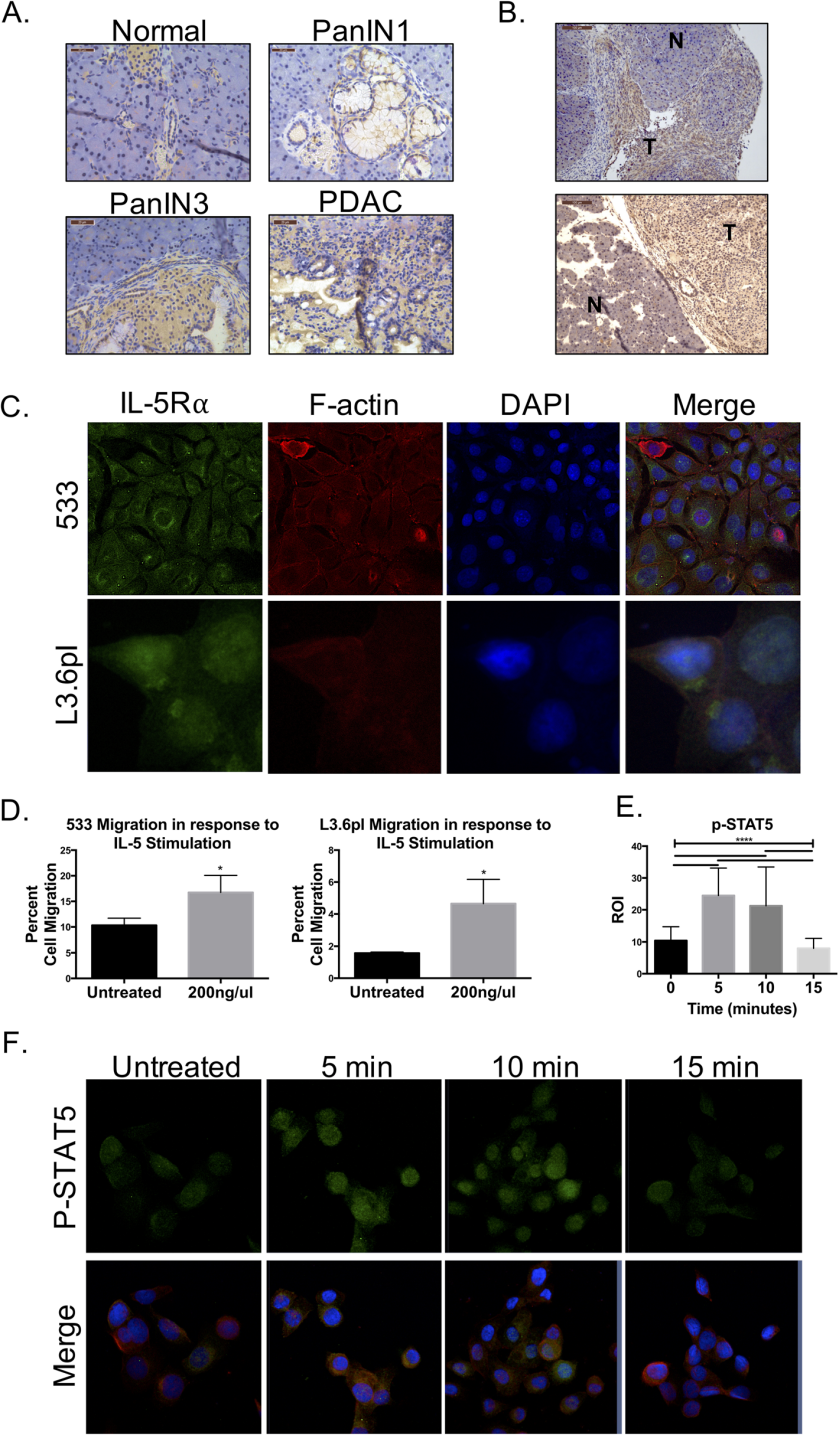
IL-5Rα signaling activates Stat5 and promotes migration in PDAC cells. **a** Immunohistochemistry for IL-5Rα in increasing stages of pancreatic cancer in genetically modified mouse model (40x images). **b** Immunohistochemistry for IL-5Rα on tumors developed from orthotopically transplanted murine Panc02 (top) and human L3.6pl PDAC cells (bottom). **c** Immunofluorescent staining and confocal imaging for IL-5Rα (green), phalloiden (red), and nucleus (DAPI, blue). **c** Transwell assay analysis of total percent migration of 533 and L3.6pl cells in the presence of soluble IL-5 (0 or 200 ng/mL) (*n* = 3). **d** Confocal microscopy and MFI quantification for phospho-Stat5 (green) in L3.6pl cells treated with IL-5 for 5 or 15 min. Red phalloidin and blue DAPI shown in merged images (63x)

IL-5R*α* is usually found on both eosinophils and B-cells for activation through IL-5 stimulus, and its expression has yet to be reported on pancreatic tissue. To determine endogenous expression of IL-5R*α* we analyzed tissues from our genetically modified mouse models and PDAC tumor cells lines. Here we report that IL-5R*α* was weakly expressed during early stages of mouse PDAC initiation, but expression was increased in PanIN3 and PDAC lesions (Fig. 4a). This is consistent with results from PDAC cell lines, including two derived from different Akt1^Myr^/KRas^G12D^ mice (533 shown and 9C not shown) [25], and another from a highly metastatic human tumor (L3.6pl). In accordance with prior publications [34–36], IL-5R*α* was expressed on the cell membrane, in the cytosol and perinuclear (Fig. 4c). Also, tumorigenic human L3.6pl and murine Panc02 pancreatic tumor cells retained expression of IL-5Rα when orthotopically injected into NSG mice or C57Bl6 mice, respectively (Fig. 4b).

To determine if the IL-5R pathway can be activated by IL-5, we looked at downstream signaling of STAT5 and migration. In the presence of soluble IL-5, murine 533 and human L3.6pl cell migration increased 1.7 and 3.5 fold, respectfully, compared to the untreated control (Fig. 4c). This increase in cell migration was not an artifact from increased proliferation. MTS assay results showed there was not a significant increase in the number of 533 or L3.6pl cells after 48 h of IL-5 ligand stimulation (data not shown). Activation of Stat5, a classical downstream effector of IL-5Rα in eosinophils [37], was significantly up-regulated and localized to the nucleus in PDAC cells after 5 min of IL-5 stimulation, then reduced to transient levels at 15 min (Fig. 4e-f). These results are supportive of a prior study highlighting IL-5 as a mediator for migration and invasion in bladder cancer through MMP-9 activity and Stat signaling [38]. The finding of activation of STAT5 in pancreatic tumors is also consistent with previous reports that suggest it is a molecular target in pancreatic cancer, as it is expressed in 50% of human PDAC [39].

### Expression of IL-5 Rα in human PDAC

**Fig. 5:**
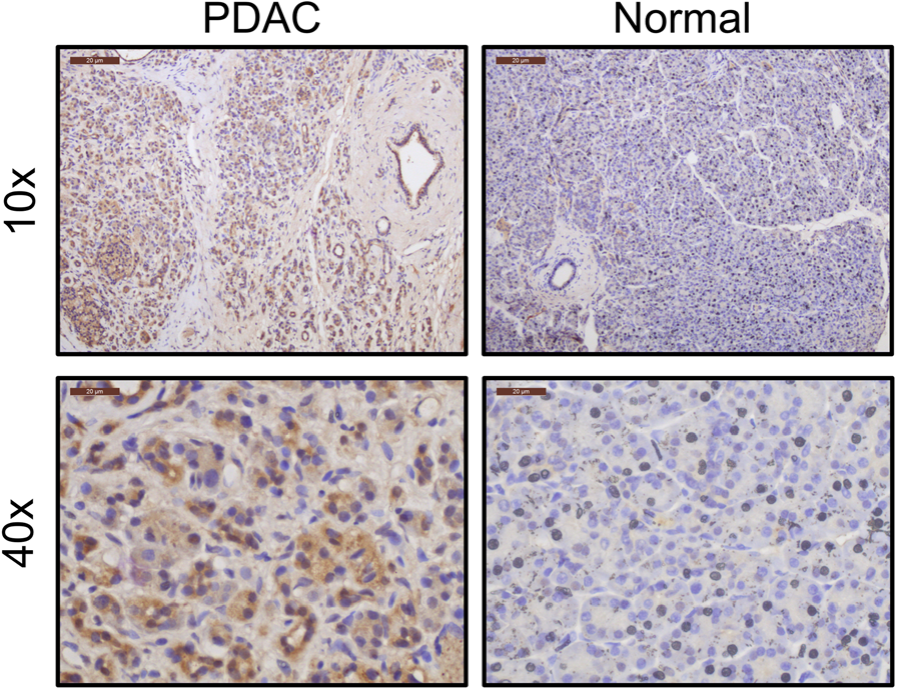
IL-5Rα is expressed in human pancreatic cancer. Immunohistochemistry for IL-5Rα in human pancreatic cancer in patient samples

IL-5Rα was detected by immunohistochemistry in human PDAC arrays, further supporting a role in PDAC tumorigenesis (Fig. 5). All seven patients with confirmed diagnosis of PDAC stained positively with antibody detection for IL-5Rα. Two ampullary carcinomas, two cholangiocarcinomas, a benign case of pancreatitis and a benign bile duct adenoma resulted in negative detection of IL-5Rα, supporting a specificity for cancerous pancreatic ductal cells. Patient information and pathology diagnosis is outlined in supplemental Table 2.

## Discussion

Herein, we present a detailed description of changes to the pancreatic microenvironment and leukocyte infiltration identified using genetic mouse models. Primarily, we found that Akt1^Myr^/KRas^G12D^ mice exhibited accentuated severe chronic pancreatitis when injected with cerulein, which led to accelerated pancreatic tumor progression. As a component of the chronic inflammation, we found increased M2 response was detected in mice with fibrotic disease, which corresponded to an increased T_h_2 associated infiltration of granulocytes and B-cells. Our observations are consistent with previous studies of eosinophil function in fibrotic disease tissues and their influence in regulating a T_h_2-immune response [12, 13].

Eosinophils have been proposed as pivotal to several aspects of tissue remodeling by activating fibroblasts to increase collagen production and fibrosis [40]. In this study, eosinophils were found in increasing numbers in regions of desmoplasia in both experimentally induced chronic pancreatitis and human stage II PDAC. Accumulation of eosinophils and mast cells have been noted in both pancreatitis and malignant lesions [15, 22]. A previous report has shown that eosinophils may provide a tumor-killing role in some solid tumors, however there is a gap in knowledge regarding the role of eosinophils in pancreatic cancer [8]. Infiltrating eosinophils lacked a cytotoxic receptor, NKG2D, in mice exhibiting fibrosis. In contrast, eosinophils express NKG2D in tissues with focal and diffuse pancreatitis. These findings provide evidence to support the assertion that desmoplasia may provide a unique niche conducive to polarizing eosinophils towards a tumor promoting phenotype. This study also corroborates previous reports of perilobular and interacinar periostin upregulation in chronic pancreatitis [41]. Periostin has been reported to increase eosinophil migration and activation, and may be a mechanism that facilitates an increased number of eosinophils in chronic pancreatitis [42, 43].

A second mechanism for eosinophil recruitment in PDAC may be in response to local secretion of endogenous IL-5 from damaged acinar tissue during pancreatitis. Results here suggest that there is a low level of IL-5 expression in normal pancreatic cells, but IL-5 expression increases during acute inflammation in cells that have undergone ADM. IL-5 expression is downregulated after cells have undergone transformation to PanIN precursor lesions.

We found that, PanIN lesions and PDAC cells express increased levels of IL-5Rα, and PDAC cells have increased migration and STAT5 activation in the presence of soluble IL-5. STAT5 activation increases chemotherapeutic resistance [44] while its inhibition in PDAC is associated with reduced tumor growth and metastasis [39]. Therefore, IL-5Rα and its downstream activation of oncogenic STAT5 signaling may provide a strategy for novel molecular therapeutics. More studies are needed to delineate the regulation of IL5Rα and STAT5-activation- mediated induction of migration in pancreatic tumor cells.

**Fig. 6:**
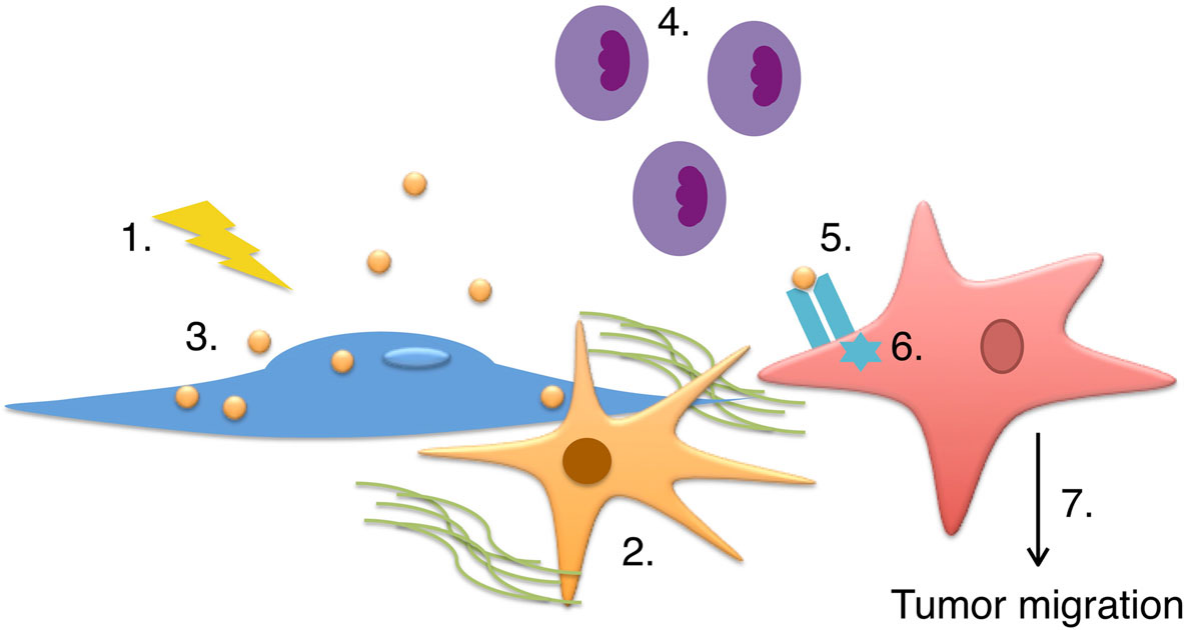
Proposed mechanism for IL-5 secretion and tumor migration. (1) Upon inflammation (2) pancreatic stellate cells become activated and secrete collagen and periostin. (3) Acinar cells begin to undergo acinar-to-ductal metaplasia and secrete IL-5, (4) which recruits eosinophils into the microenvironment. Eosinophils are retained in the microenvironment by periostin secreted pancreatic stellate cells. Prolonged inflammation further converts ADM cells into PanINs. PanIN lesions and pancreatic tumor cells express IL-5 receptor. (5) When IL-5 receptor is bound by IL-5, (6) STAT 5 signaling pathway is activated which leads to (7) tumor cell migration by a possible MMP dependent mechanism

We postulate that acinar damage in response to inflammation can cause the release of IL-5 into the microenvironment, which in turn helps to recruit eosinophils and B-cells into the microenvironment. Concurrently, increased periostin deposition results in the accumulation and retention of immune cells such as eosinophils in the stromal regions. This prolonged Th2-associated immune response helps to perpetuate the fibrotic buildup as well as tumor development (Fig. 6). Overall, cytokine/ receptor switching in pre-neoplastic lesions might be a novel mechanism for how cells adapt their expression of cytokine receptors to utilize the microenvironment for malignant progression.

## Conclusions

Chronic inflammation induces increased pancreatic cancer progression, in part through the attraction of immune cells such as eosinophils to areas of activated fibrosis. Results suggest that pancreas localized IL-5 stimulates the increase of IL-5Rα on ductal tumor cells and increases pancreatic tumor motility. Collectively, IL-5/IL-5Rα signaling in the pancreatic tumor microenvironment is a novel mechanism to facilitate tumor progression.

## Supplementary information

**Additional file 1: Figure S1.** Oncogenic Akt1^myr^ alone does not induce stromal changes and increased immune cell infiltration with cerulein injections. **Table S1.** Akt1^Myr^/KRas^G12D^ mice with chronic inflammation progresses to more severe pancreatic cancer and metastasis compared to KRas^G12D^ mice. **Figure S2.** Immune cell infiltration in pancreatic cancer. **Figure S3.** Gating strategy for identification of M1 and M2 macrophage populations. **Figure S4.** Gating strategy for identification of cytotoxic and non-cytotoxic eosinophil populations. **Table S2.** Patient information and pathology for tissue samples evaluated for IL-5Rα.

## Data Availability

Data sharing is not applicable to this article as no datasets were generated or analyzed during the current study.

## Abbreviations

IL: Interleukin
PDAC: Pancreatic ductal adenocarcinoma
ADM: Acinar-to-ductal metaplasia
PSC: Pancreatic stellate cell
EPO: Eosinophil peroxidase
TATE: Tumor-associated tissue eosinophilia
Th: T-helper
TNF: Tumor Necrosis Factor
MIP: Macrophage inflammatory protein
CER: Cerulein
Wt: Wild type
H&E: Hematoxylin and eosin
MFI: Mean fluorescent intensity
PBS: Phosphate buffered saline
SEM: Standard error of the mean
SD: Standard deviation
PanIN: Pancreatic Intraepithelial Neoplasia
SMA: Smooth muscle actin
MHC: Major histocompatibility complex
C.E.M.: Combined Eosinophil and Mast Cell
MTS: 3-(4,5-dimethylthiazol-2-yl)-5-(3-carboxymethoxyphenyl)-2-(4-sulfophenyl)-2H-tetrazolium)
MMP-9: Matrix metallopeptidase 9
