# Supplementary material for: Identification of a novel IL-5 signaling pathway in chronic pancreatitis and crosstalk with pancreatic tumor cells
Source: Cell Commun Signal. 2020 Jun 17;18:95. doi: 10.1186/s12964-020-00594-x (PMC7302008; doi:10.1186/s12964-020-00594-x)
Supplement: Supplementary file 2 — Additional file 1: Figure S1. Oncogenic Akt1myr alone does not induce stromal changes and increased immune cell infiltration with cerulein injections. Table S1. Akt1Myr/KRasG12D mice with chronic inflammation progresses to more severe pancreatic cancer and metastasis compared to KRasG12D mice. Figure S2. Immune cell infiltration in pancreatic cancer. Figure S3. Gating strategy for identification of M1 and M2 macrophage populations. Figure S4. Gating strategy for identification of cytotoxic and non-cytotoxic eosinophil populations. Table S2. Patient information and pathology for tissue samples evaluated for IL-5Rα. [file 12964_2020_594_MOESM2_ESM.zip › Supplemental Figure Legends.docx]

**Supplemental Figure Legends**

**Figure S.1 Oncogenic Akt1^myr^ alone does not induce stromal changes and increased immune cell infiltration with cerulein injections.** (A) Hematoxylin and eosin staining of the pancreas for Akt (top panel) and Wt (bottom panel) mice injected with 4 rounds of CER injections (4-month-old), 8 rounds of CER injections (6-month-old), or PBS controls. Images were acquired at 10x magnification and the scale bar denotes 100μm. (B) Flow cytometry results of immune cells collected from perfused pancreas of Akt and Wt mice after 4 rounds of CER or PBS as a comparative baseline. Total lymphocytes were gated first by forward and side scatter then for total lymphocyte marker CD45. Individual cell populations were then sub-gated according to CD11b+ (macrophages and monocytes), CD3+ (T-cells), and CD45R (B-cells). A two-way ANOVA and a Tukey’s multiple comparison analyze statistical significance between means.

**Table S.1: Akt1^Myr^/KRas^G12D^** **mice with chronic inflammation progresses to more severe pancreatic cancer and metastasis compared to KRas^G12D^ mice.** Acute focal inflammation was scored by defining the presence of neutrophils (IHC staining) located in and around the ducts. Chronic inflammation was determined by fibrosis (collagen staining) and increased immune cell infiltration. Pancreatic Intraepithelial Neoplasia (PanIN), PDAC, and metastasis were scored using defined cellular and structural changes in the ductal tissue.

**Figure S.2 Immune cell infiltration in pancreatic cancer.** (A) Histological comparison of normal tissue changes due to genetic alterations in pancreatic tissue from Akt1^Myr^/KRas^G12D^ and KRas^G12D^ mice with PBS injections at age 4 and 6 months of age. (B) Immunohistochemical staining for CD19 (B cells, brown) in Akt1^Myr^/KRas^G12D^ and KRas^G12D^ mice after 4 and 8 CCK injections. (C) Eosinophils (C.E.M., red) staining in Akt1^Myr^/KRas^G12D^ and KRas^G12D^ mice after 8 CCK injections. (D) Eosinophils (C.E.M., red) in human stage II adenocarcinoma and stage II adeno-squamous carcinoma. Black arrows indicated positive stained eosinophils.

**Figure S.3 Gating strategy for identification of M1 and M2 macrophage populations.** Lymphocyte populations were gated by analyzing the width and height of forward and side scatter, and were positive for CD45. Macrophages were gated on mature murine macrophage marker F4/80. M2 macrophages were defined as positive for membrane bound MHC class II and CD206, and negative for intracellular iNOS. M1 macrophages were defined as positive for iNOS but negative for MHC class II and CD206.

**Figure S.4 Gating strategy for identification of cytotoxic and non-cytotoxic eosinophil populations.** Lymphocyte populations were gated by analyzing the width and height of forward and side scatter. Eosinophils were gated on the innate lymphocytes marker CD11b and cytokine receptor CD193, which is highly expressed on eosinophils. CD11c+ cells were excluded and eosinophil marker SiglecF+ cells were gated for cytotoxicity. After gating, cytotoxic eosinophils are NKG2D+ and not cytotoxic eosinophils are NKG2D-.

**Table S.2 Patient information and pathology for tissue samples evaluated for IL-5Rα.** Abbreviations: diff: differentiated; IPMN: intraductal papillary mucinous neoplasm, LN: lymph node, met: metastasis, NEO: neoadjuvant therapy; PDAC: pancreatic ductal adenocarcinoma; PFS: progression free survival; sm: small
