# Supplementary material for: Identification of a novel IL-5 signaling pathway in chronic pancreatitis and crosstalk with pancreatic tumor cells
Source: Cell Commun Signal. 2020 Jun 17;18:95. doi: 10.1186/s12964-020-00594-x (PMC7302008; doi:10.1186/s12964-020-00594-x)
Supplement: Supplementary file 2 — Additional file 1: Figure S1. Oncogenic Akt1myr alone does not induce stromal changes and increased immune cell infiltration with cerulein injections. Table S1. Akt1Myr/KRasG12D mice with chronic inflammation progresses to more severe pancreatic cancer and metastasis compared to KRasG12D mice. Figure S2. Immune cell infiltration in pancreatic cancer. Figure S3. Gating strategy for identification of M1 and M2 macrophage populations. Figure S4. Gating strategy for identification of cytotoxic and non-cytotoxic eosinophil populations. Table S2. Patient information and pathology for tissue samples evaluated for IL-5Rα. [file 12964_2020_594_MOESM2_ESM.zip › Supplemental Table 2.docx]

| **IL-5 STAINING** | **TUMOR GROUP** | **GENDER** | **AGE AT SURGERY** | **PATHOLOGY GRADE** | **LN#** | **PATHOLOGY DIAGNOSIS** | **NEO ADJUVANT** |
| --- | --- | --- | --- | --- | --- | --- | --- |
| - | BENIGN | M | 84 | BENIGN | 0/16 | Benign; lymphoplasmacytic sclerosingpancreatits | NONE |
| - | BENIGN | M | 77 | Bile duct adenoma | 0/9 | Bile duct adenoma, no invasive carcinoma | NONE |
| + | PDAC | F | 65.2 | T1N0M0 stage 1 | 0/10 | PDAC; well diff; invasive arising in large IPMN with high grade despmoplasia | NONE |
| + | PDAC | M | 65.3 | T3N0M0 stage IIA | 0/6 | PDAC; extrapancreas extension; moderately diff | NEO Abraxane gemcitabine |
| + | PDAC | M | 59.9 | T1N1M0 stage IIB | 1/16 | PDAC; poorly diff; G3; noninvasive | NEO Fulfurinox, radiation; tumor responded (G3->G1) |
| + | PDAC | F | 77.9 | T3N1M0 stage IIB | 3/12 | PDAC; G3 poorly diff; invasive; chronic pancreatitis; esophageal inflammation; fungal infection | NEO Abraxane gemcitabine |
| + | PDAC | F | 77 | T3N1M0 stage IIB | 1/12 | PDAC; poorly diff | NEO |
| + | PDAC | F | 79 | T3N1M0 stage IIB | 3/34 | PDAC; poorly diff; G3; invasive found in low grade IPMN; squamous component; perineural invasion; distal pancreatic cancer | NONE |
| + | PDAC | M | 70 | T3N1M0 stage IIB | 5/16 | PDAC; well diff ductal with mucinous features; invading duodenal wall and soft tissues | NONE |
| - | PDAC | M | 65.3 | T3N1M1 stage IV with liver involvement | 5/18 | PDAC; well differentiated; met to duodenum; liver met; invasion sm bowel, ampullary, peritoneum; G3; poorly diff Bile Duct Adenocarcinoma | NEO Abraxane gemcitabine |
| - | AMPULLARY | F | 76.9 | T4N1M1 stage III ampullary | 3/8 | Mets sm bowel/peri-ampullary; G3 poorly diff; extension from LN; invasive | NEO gemcitabine Abraxane |
| - | AMPULLARY | M | 73.5 | T4N1M0 stage III ampullary | 0/3 | Poorly diff; invasive; peri-ampullary; through sm bowel; bile duct | NONE |
| - | CHOLANGIO CARCINOMA | M | 60 | T3N1M0; stage IIB | 3/20 | Bile duct tumor; poorly diff adenocarcinoma of common bile duct; invading duodenal wall, pancreas and peripancreatic adipose | NONE |
