# Supplementary figures and images for: Identification of a novel IL-5 signaling pathway in chronic pancreatitis and crosstalk with pancreatic tumor cells
Source: Cell Commun Signal. 2020 Jun 17;18:95. doi: 10.1186/s12964-020-00594-x (PMC7302008; doi:10.1186/s12964-020-00594-x)

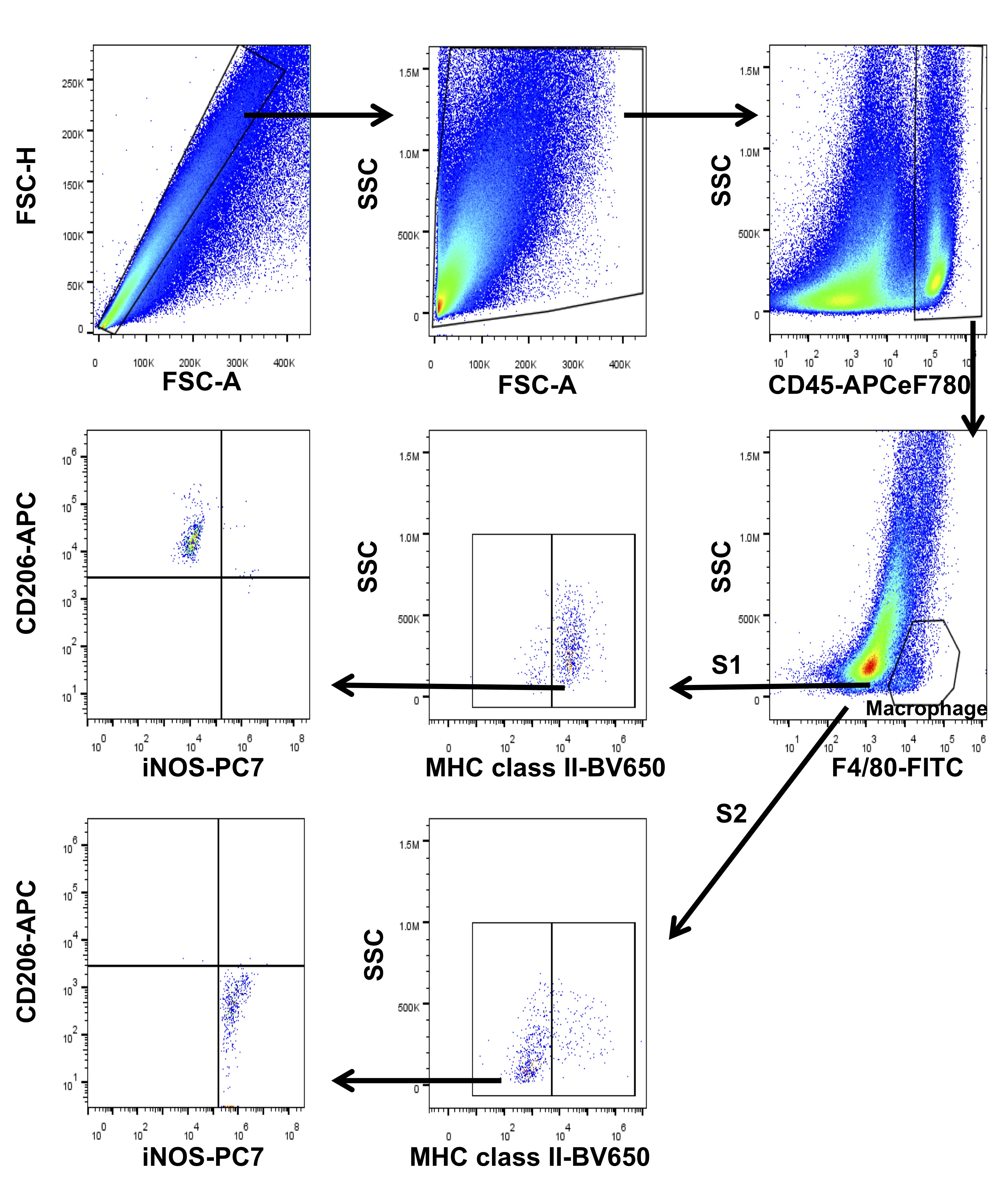

Supplement: Supplementary file 2 — Additional file 1: Figure S1. Oncogenic Akt1myr alone does not induce stromal changes and increased immune cell infiltration with cerulein injections. Table S1. Akt1Myr/KRasG12D mice with chronic inflammation progresses to more severe pancreatic cancer and metastasis compared to KRasG12D mice. Figure S2. Immune cell infiltration in pancreatic cancer. Figure S3. Gating strategy for identification of M1 and M2 macrophage populations. Figure S4. Gating strategy for identification of cytotoxic and non-cytotoxic eosinophil populations. Table S2. Patient information and pathology for tissue samples evaluated for IL-5Rα. [file 12964_2020_594_MOESM2_ESM.zip › Suppl Figure 3.jpg]

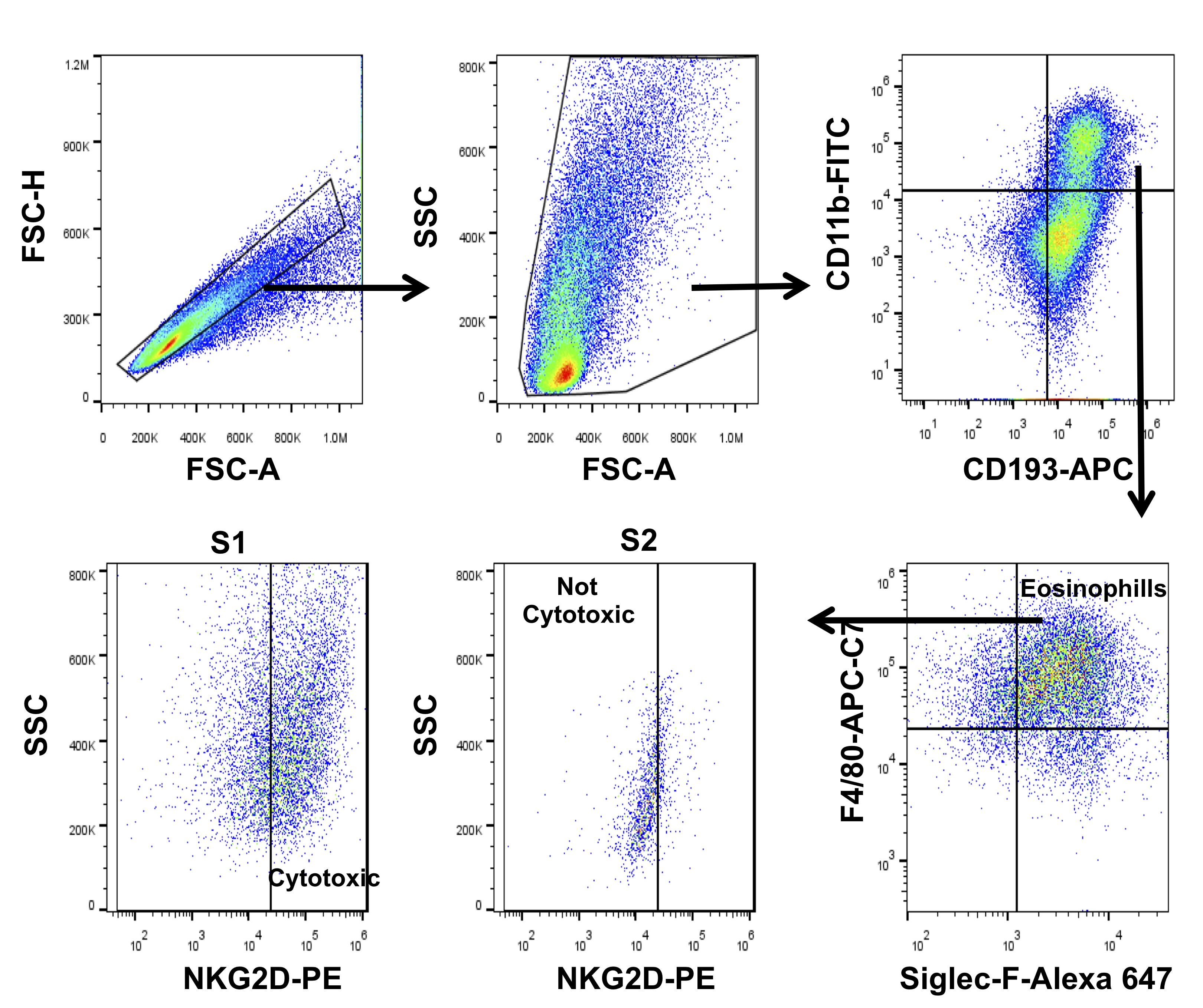

Supplement: Supplementary file 2 — Additional file 1: Figure S1. Oncogenic Akt1myr alone does not induce stromal changes and increased immune cell infiltration with cerulein injections. Table S1. Akt1Myr/KRasG12D mice with chronic inflammation progresses to more severe pancreatic cancer and metastasis compared to KRasG12D mice. Figure S2. Immune cell infiltration in pancreatic cancer. Figure S3. Gating strategy for identification of M1 and M2 macrophage populations. Figure S4. Gating strategy for identification of cytotoxic and non-cytotoxic eosinophil populations. Table S2. Patient information and pathology for tissue samples evaluated for IL-5Rα. [file 12964_2020_594_MOESM2_ESM.zip › Suppl Figure 4.jpg]

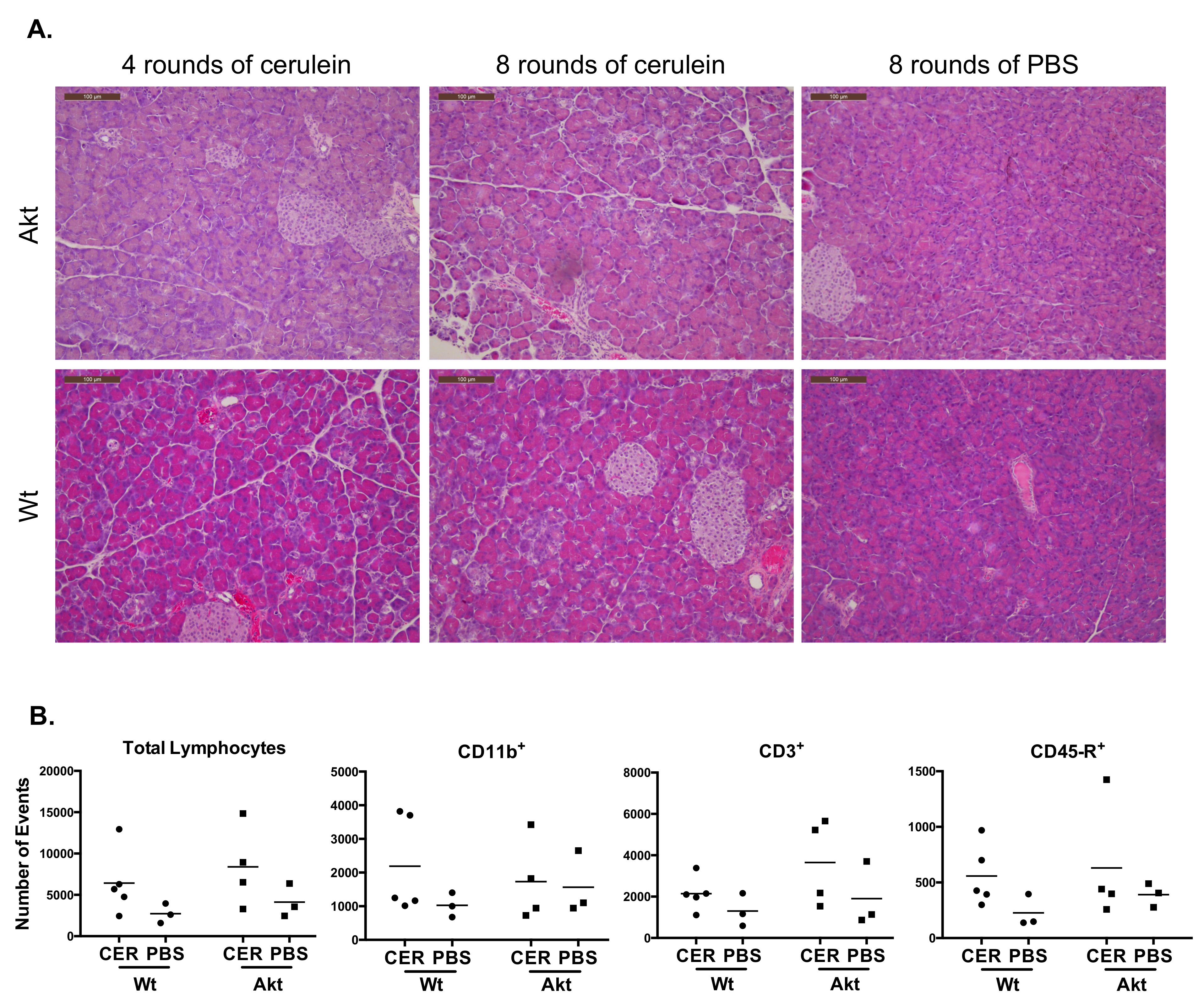

Supplement: Supplementary file 2 — Additional file 1: Figure S1. Oncogenic Akt1myr alone does not induce stromal changes and increased immune cell infiltration with cerulein injections. Table S1. Akt1Myr/KRasG12D mice with chronic inflammation progresses to more severe pancreatic cancer and metastasis compared to KRasG12D mice. Figure S2. Immune cell infiltration in pancreatic cancer. Figure S3. Gating strategy for identification of M1 and M2 macrophage populations. Figure S4. Gating strategy for identification of cytotoxic and non-cytotoxic eosinophil populations. Table S2. Patient information and pathology for tissue samples evaluated for IL-5Rα. [file 12964_2020_594_MOESM2_ESM.zip › Supple Figure 1.jpg]

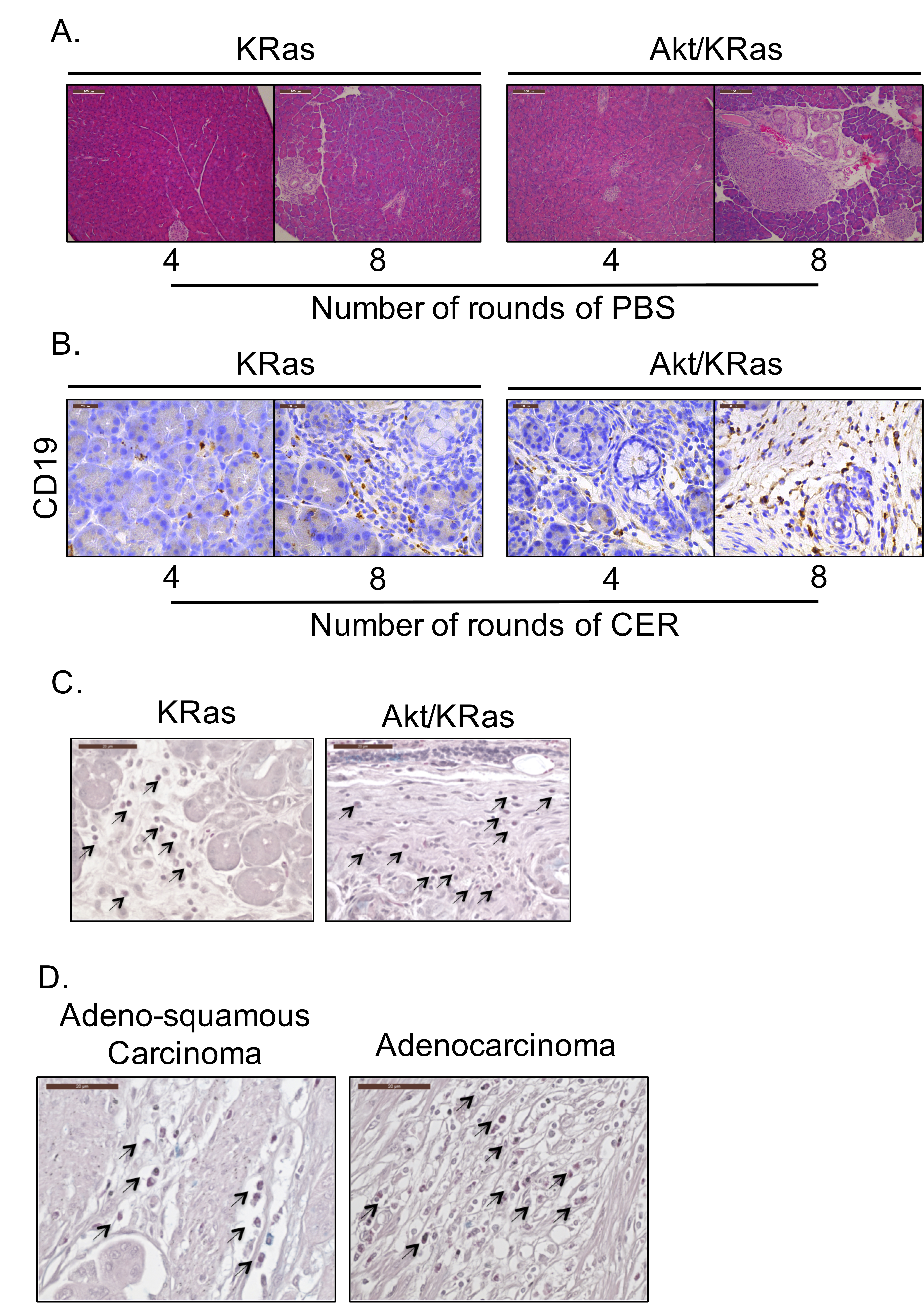

Supplement: Supplementary file 2 — Additional file 1: Figure S1. Oncogenic Akt1myr alone does not induce stromal changes and increased immune cell infiltration with cerulein injections. Table S1. Akt1Myr/KRasG12D mice with chronic inflammation progresses to more severe pancreatic cancer and metastasis compared to KRasG12D mice. Figure S2. Immune cell infiltration in pancreatic cancer. Figure S3. Gating strategy for identification of M1 and M2 macrophage populations. Figure S4. Gating strategy for identification of cytotoxic and non-cytotoxic eosinophil populations. Table S2. Patient information and pathology for tissue samples evaluated for IL-5Rα. [file 12964_2020_594_MOESM2_ESM.zip › Figure S2.jpg]
